# Coronary Calcium Scoring with First Generation Dual-Source Photon-Counting CT—First Evidence from Phantom and In-Vivo Scans

**DOI:** 10.3390/diagnostics11091708

**Published:** 2021-09-18

**Authors:** Matthias Eberhard, Victor Mergen, Kai Higashigaito, Thomas Allmendinger, Robert Manka, Thomas Flohr, Bernhard Schmidt, Andre Euler, Hatem Alkadhi

**Affiliations:** 1 Institute of Diagnostic and Interventional Radiology, University Hospital Zurich, University of Zurich, 8091 Zurich, Switzerland; Victor.mergen@usz.ch (V.M.); kai.higashigaito@usz.ch (K.H.); robert.manka@usz.ch (R.M.); andre.euler@usz.ch (A.E.); hatem.alkadhi@usz.ch (H.A.); 2 Siemens Healthineers, 91301 Forchheim, Germany; thomas.allmendinger@siemens-healthineers.com (T.A.); thomas.flohr@siemens-healthineers.com (T.F.); bernhard.schmidt@siemens-healthineers.com (B.S.); 3 Department of Cardiology, University Heart Center, University Hospital Zurich, University of Zurich, 8091 Zurich, Switzerland

**Keywords:** agatston score, computed tomography, coronary CT angiography, coronary calcium scoring, photon counting computed tomography, virtual monoenergetic imaging

## Abstract

We evaluated the accuracy of coronary artery calcium (CAC) scoring on a dual-source photon-counting detector CT (PCD-CT). An anthropomorphic chest phantom underwent ECG-gated sequential scanning on a PCD-CT at 120 kV with four radiation dose levels (CTDIvol, 2.0–8.6 mGy). Polychromatic images at 120 kV (T3D) and virtual monoenergetic images (VMI), from 60 to 75 keV without quantum iterative reconstruction (no QIR) and QIR strength levels 1–4, were reconstructed. For reference, the same phantom was scanned on a conventional energy-integrating detector CT (120 kV; filtered back projection) at identical radiation doses. CAC scoring in 20 patients with PCD-CT (120 kV; no QIR and QIR 1–4) were included. In the phantom, there were no differences between CAC scores of different radiation doses (all, *p* > 0.05). Images with 70 keV, no QIR (CAC score, 649); 65 keV, QIR 3 (656); 65 keV; QIR4 (648) and T3D, QIR4 (656) showed a <1% deviation to the reference (653). CAC scores significantly decreased at increasing QIR levels (all, *p* < 0.001) and for each 5 keV-increase (all, *p* < 0.001). Patient data (median CAC score: 86 [inter-quartile range: 38–978] at 70 keV) confirmed relationships and differences between reconstructions from the phantom. First phantom and in-vivo experience with a clinical dual-source PCD-CT system shows accurate CAC scoring with VMI reconstructions at different radiation dose levels.

## 1. Introduction

Cardiovascular risk assessment is crucial for the prevention of adverse cardiovascular events [1,2,3]. Compared to well established clinical risk scores [4,5,6], the coronary artery calcium scoring (CAC) score provides incremental prognostic information predicting the risk of future events.

Agatston et al. [7] described the methodology of CAC scoring using electron beam CT (EBCT). Meanwhile, EBCT has been surpassed by helical energy-integrating detector CT (EID-CT) [8]. Here, the standardized technique of CAC scoring using ECG-gated non-contrast CT with a tube voltage of 120 kV and filtered back projection (FBP) emerged as the validated standard [7,9], enabling reproducible CAC scoring with different CT systems [10].

Compared to EID-CT, photon-counting detector CT (PCD-CT) represents a fundamentally different approach for CT imaging [11,12,13]. Photon-counting detectors count the number of incoming photons and the electronic signal is proportional to the deposited energy of each photon [11]. PCD-CT has the potential benefit of a higher contrast-to-noise ratio for high atomic number materials such as iodine or calcium, improved spatial resolution, and reduction of calcium blooming [11,14]. Recently, the first whole-body, full field-of-view dual-source PCD-CT system was cleared for clinical use. With this PCD-CT scanner, the new standard of post-processing is virtual monoenergetic images (VMI), which are directly and automatically generated by the scanner. Along with this, a new iterative image reconstruction algorithm called Quantum Iterative Reconstruction (QIR) was introduced.

The purpose of our study was to evaluate the accuracy of CAC scoring on first generation dual-source PCD-CT and to determine the optimal VMI keV level and best iterative reconstruction technique at different radiation doses in a phantom and in patients.

## 2. Methods

### 2.1. Study Population

This work comprised a phantom study and in-vivo data of 20 consecutive patients with coronary calcifications undergoing an ECG-triggered non-enhanced CT scan for quantification of coronary calcifications between April–May 2021. This single-center study was approved by the local ethics committee; written informed consent was obtained from all patients. The study was performed in compliance with the Declaration of Helsinki.

### 2.2. Phantom Study

#### 2.2.1. Anthropomorphic Phantom

We used a commercially available 30 cm diameter anthropomorphic chest phantom with a 10 cm diameter insert for calcium scoring (QRM, Moehrendorf, Germany). The phantom contains nine cylindrical inserts varying in size (1 mm, 3 mm and 5 mm) and density (hydroxyapatite at 200 mg/cm^3^, 400 mg/cm^3^ and 800 mg/cm^3^) [10]. For this study, the manufacturer optimized the phantom for spectral imaging by ensuring a constant background material value of 35 Hounsfield Unit (HU) at all energy thresholds acquired by the PCD-CT system.

#### 2.2.2. Phantom Scan Acquisition

ECG-triggered sequential acquisitions were performed on a first-generation PCD-CT system with Quantum Technology (NAEOTOM Alpha; Siemens Healthineers; Forchheim, Germany) equipped with two photon-counting detectors (cadmium telluride), each with a 144 × 0.4 mm collimation. The tube voltage was 120 kV in the multi-energy (QuantumPlus) mode. Tube current time product was adjusted according to four different image quality (IQ) levels (20, 40, 60 and 80). The IQ level is a novel parameter that represents quality reference mAs, denoting effective mAs applied for the protocol specific reference water-equivalent diameter with a CT geometry correction, particularly for the effect of the focal spot to iso-center distance. Therefore, the image quality level provides a system and reconstruction-independent image quality definition. The ECG-triggered window was set to a minimal width enabling reconstruction at a single phase of 75% using an artificial ECG signal with 60 bpm. Scan parameters resulted in a volume CT dose index (CTDI_vol_) of 2.0, 3.8, 6.4 and 8.6 mGy, respectively.

#### 2.2.3. Scan Reconstruction

VMI were reconstructed at 75 keV, 70 keV, 65 keV and 60 keV. In addition, conventional images using the data of the lowest threshold (20 keV), being comparable to polychromatic 120 kV images, were reconstructed (hereafter called T3D). Furthermore, images were reconstructed with a spectral reconstruction either with QIR = off (hereafter called *FBP*), which is comparable to a conventional reconstruction in terms of the expected noise level and with QIR at all four strength levels (QIR 1–4), resulting in 25 different sets of images. For all monochromatic reconstructions, the algorithm utilizes the full spectral information of the counting detector data to iteratively correct for beam-hardening artefacts and geometric cone-beam artefacts in combination with a prior based denoising algorithm, which is synchronized across the contributing threshold data sets. At QIR strength levels 1 (lowest level) to 4 (highest level), additional iterative noise reduction is performed. The slice thickness was 3 mm, the increment 1.5 mm and the same reconstruction kernel was used (Qr36) for all data.

#### 2.2.4. Reference

For reference, the same chest phantom was scanned four times in the sequential mode on a single-source EID-CT (SOMATOM Edge Plus; Siemens Healthineers; Forchheim, Germany) at 120 kV. The tube current-time product was adjusted to achieve an identical CTDI_vol_ of 2.0, 3.8, 6.4, and 8.6 mGy as with PCD-CT. Images were reconstructed as follows: FBP, Sa36 kernel, 3 mm slice thickness, and 1.5 mm increment.

### 2.3. Patient Scans

**Figure 1:**
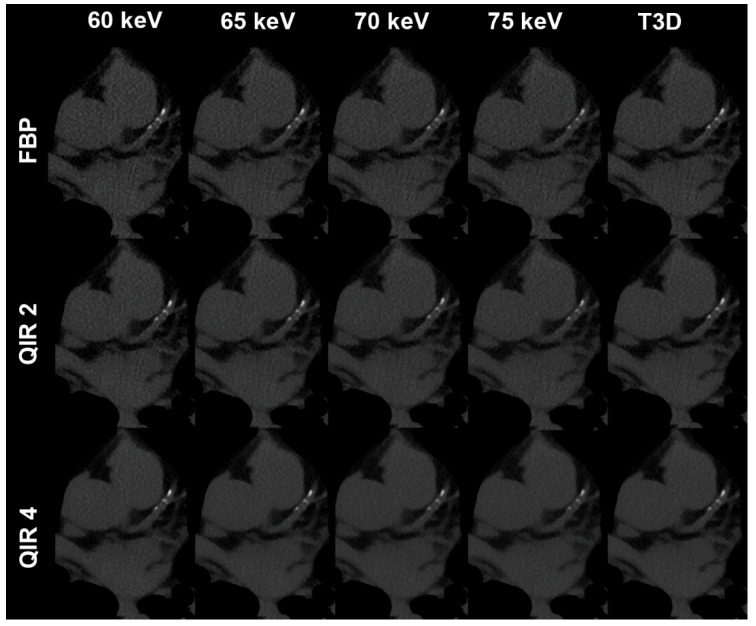
Representative images of a 56-year-old male patient undergoing non-enhanced ECG-gated CT dedicated to coronary calcium scoring. Transverse CT image data sets at the same slice positions reconstructed with 60 keV (far left column), 65 keV (second to left column), 70 keV (middle column), 75 keV (second to right column) and T3D (polychromatic 120 kV reconstruction; far right column); filtered back projection (upper row) and Quantum Iterative Reconstruction at strength levels 2 (middle row) and 4 (bottom row). Coronary calcium scores decrease with increasing keV levels (lowest scores for 75 keV) and with increasing strength of QIR (lowest scores for QIR level 4). T3D reconstructions were similar to 65 keV reconstructions.

Scans were performed in the sequential mode on a first generation PCD-CT. Tube voltage was 120 kV, the tube current-time product was set at an IQ level of 20, using automated tube current modulation. Reconstructions were identical as in the phantom (Exemplary image on Figure 1).

### 2.4. Coronary Artery Calcium Scoring

One reader (with 8 years of experience in cardiovascular radiology) quantified the CAC score using a commercially available software (syngo.via VB60, Siemens Healthineers; Forchheim, Germany) as previously described [7,15].

### 2.5. Statistical Analysis

Continuous variables were expressed as mean ± standard deviation or median and inter-quartile range, where appropriate. Categorical variables are presented as count and percentages. Agatston scores at different dose levels (IQ levels) and reconstruction algorithms were compared using the Friedman test with Bonferroni correction. The relative percentage error was calculated as follows: 100 × (CAC score[PCD-CT] − CAC score[EID-CT])/(CAC score[EID-CT]).

*p* < 0.05 was considered to indicate statistical significance. Analyses were performed with commercial software (SPSS, Chicago, IL, USA).

## 3. Results

### 3.1. Phantom Study

**Figure 2:**
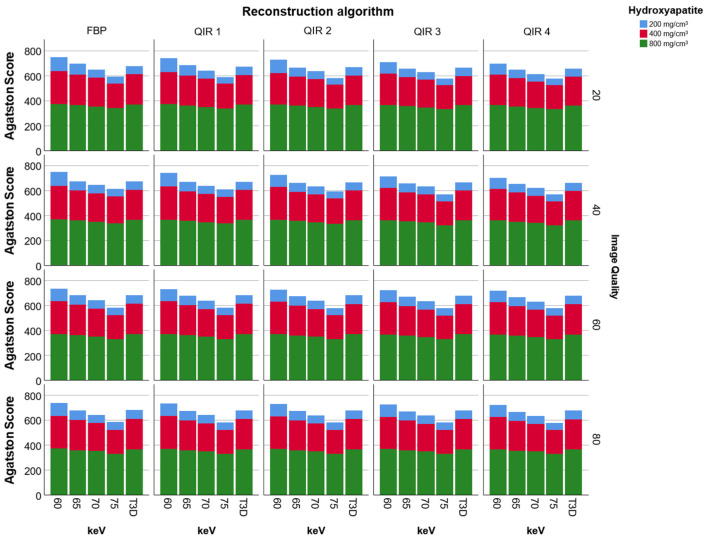
Data from scans of an anthropomorphic chest phantom with an insert for calcium scoring shows that virtual monoenergetic image reconstructions with increasing keV (*p* < 0.001 for each 5 keV increase) and increasing strength levels of Quantum Iterative Reconstruction (QIR; *p* < 0.001 for each increase in strength level) lead to decreasing calcium scores. There was no difference between calcium scores of 65 keV monoenergetic and T3D (equivalent to polychromatic 120 kV) reconstructions (*p* > 0.05). There was no difference of calcium scores between various degrees of image quality and hence, radiation dose (all, *p* > 0.05).

There were no differences between CAC scores of the IQ20 scan compared to the IQ40 (*p* = 1.0), IQ60 (*p* = 1.0) and IQ80 (*p* = 0.38) scan (Figure 2). We therefore report only results of the IQ20 scan. CAC scores ranged between 574 (75 keV, QIR level 4) and 747 (60 keV, FBP).

**Figure 3:**
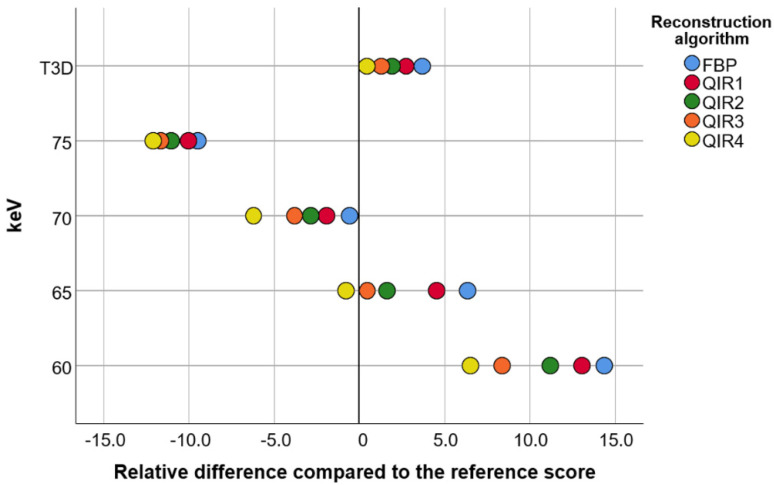
Data from a photon-counting detector CT scan of an anthropomorphic chest phantom with an insert for calcium scoring. The calcium score of image reconstructions with 65 to 75 keV as well as with T3D (and Quantum Iterative Reconstruction—QIR—at various strength levels) range within 10% of the reference score acquired with an energy-integrating detector CT. Image reconstruction with 70 keV and FBP, 65 keV and Quantum Iterative Reconstruction (QIR) at a strength level of 3 or 4 as well as with T3D and QIR at strength level 4 results in a deviation of <1% from the reference score. The lower the keV levels, the wider the distribution of calcium scores between QIR reconstructions at various strength levels.

Several image reconstructions were close to the reference CAC score (obtained with an EID-CT) of 653 (Figure 3). Image reconstructions with 65 keV and QIR level 3 (CAC Score: 656), 65 keV and QIR level 4 (CAC Score: 648), T3D and QIR level 4 (CAC Score: 656) as well as 70 keV and FBP (CAC Score: 649) all had a relative error <1% compared to the reference.

Overall, for all VMI, CAC scores were highest for image reconstructions with FBP compared to QIR. CAC scores for image reconstruction at strength level 1 were significantly lower compared to image reconstruction with FBP and CAC scores significantly decreased at each increasing level of QIR compared to the lower strength level of QIR (*p* < 0.01 for each comparison; Figure 2). For VMI, highest scores were found at 60 keV, with significantly decreasing scores at each 5 keV-increase (*p* < 0.01 for each comparison; lowest scores at 75 keV). CAC scores were not different between image reconstructions with T3D and 65 keV (*p* = 0.71; Figure 2 and Figure 3), whereas CAC scores on T3D reconstructions were significantly different to all other VMI (all, *p* < 0.001).

### 3.2. Patient Study

Twelve of the 20 patients (60%) were female, mean age was 69 ± 12 years, mean body mass index was 27.0 ± 4.7 kg/m^2^. The effective radiation dose was 0.67 ± 0.26 mSv (Table 1).

**Figure 4:**
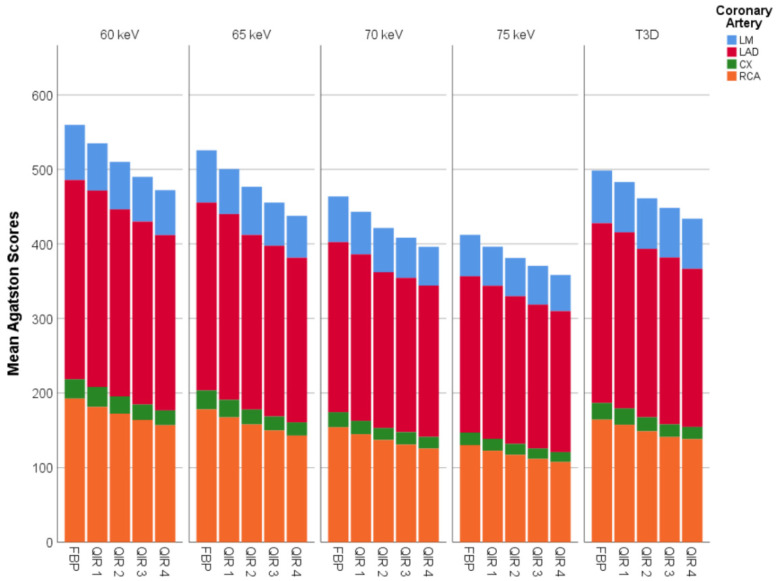
Data of 20 patients show that virtual monoenergetic image reconstructions at increasing keV (*p* < 0.001 for each eV increase) and increasing strength levels of Quantum Iterative Reconstruction (QIR; *p* < 0.001 for each increase in strength level) led to decreasing calcium scores. There was no difference of calcium scores between 65 keV monoenergetic reconstructions and T3D (equivalent to polychromatic 120 kV) (all, *p* > 0.05).

The median score of patients varied between 107 (IQR: 48–1060; 60 keV and FBP) and 49 (IQR: 20–803; 75 keV and QIR level 4; Table 2). Similar to the phantom, CAC scores were higher for image reconstructions with FBP compared to QIR for all VMI. CAC scores for image reconstruction at strength level 1 were significantly lower compared to image reconstruction with FBP (*p* < 0.01). CAC scores significantly decreased at each increasing level of QIR compared to the lower strength level of QIR (*p* < 0.01 for each comparison; Figure 4). For VMI highest scores were found at 60 keV, with significantly decreasing scores at each 5 keV-increase (all, *p* < 0.01; lowest scores at 75 keV). CAC scores were similar between T3D and 65 keV (*p* = 0.89), whereas CAC scores on T3D were significantly different to all other VMI (all, *p* < 0.001).

## 4. Discussion

CAC scoring for risk prediction of adverse cardiovascular events requires accuracy and reproducibility of measurements across various CT scanners and techniques. Our first phantom and in-vivo experience suggests that CAC scoring on a clinical dual-source PCD-CT is accurate, with a deviation of below 1% compared to the reference. Results were stable across various radiation doses. Reconstructions at increasing strength levels of QIR and with increasing keV result in decreasing CAC scores.

The Agatston score is highly dependent on the maximum attenuation of a calcified plaque as the factor used for calculation is selected according to fixed HU thresholds [7,9,17]. As the tube potential affects the energy spectra of the x-ray beam and therefore the attenuation of tissue, scan acquisitions using a tube potential of 120 kV are commonly applied to ensure comparability of CAC scores between different EID-CT systems [10]. Recently, Tao et al. proposed a kV-independent protocol for CAC scoring using a dedicated reconstruction kernel [18,19], allowing for consistent attenuation of calcifications at tube potentials other than 120 kV. Several studies evaluated iterative reconstruction algorithms to facilitate radiation dose reduction for calcium scoring scans [20,21,22,23]. However, these are usually not applied because of a systematic underestimation of calcification scores [20,21].

Post-processing with PCD-CT behaves differently compared EID-CT [11]. Due to the increased weighting of low energy x-ray photons in PCD-CT compared to EID-CT, T3D image reconstructions—providing an equivalent to conventional polychromatic 120 kV reconstructions—do not result in attenuation identical to 120 kV on EID-CT. With PCD-CT, the new standard for image post-processing is VMI. Our data show that image reconstructions on PCD-CT allow for CAC scoring with a deviation of below 1% compared to a conventional EID-CT. This was achieved using VMI at 70 keV without QIR or at 65 keV or T3D reconstructions, the latter both with QIR level 3 and/or 4.

Phantom scans at four different radiation doses showed no differences between CAC scores at high (IQ level 80) and low radiation dose (IQ level 20) scans. Therefore, we propose low radiation dose scans for calcium scoring also with PCD-CT. The possibility to use VMI reconstructions for CAC scoring opens the potential for kV-independent CAC scoring on PCD-CT, which should be subject of future studies as this may facilitate further radiation dose reduction.

We acknowledge the following study limitations. First, there is no gold standard reference for the Agatston score. However, we compared the results of the PCD-CT scanner with the results of a state-of-the-art EID-CT scanner. Second, we did not assess inter-scan reproducibility of phantom and patient scans. Symons et al. previously reported a significantly higher reproducibility of CAC scores in non-ECG gated scans of an ex-vivo heart and better agreement between standard and low dose CAC scores in non-ECG-gated scans for the PCD-CT compared to an EID-CT [24]. Third, phantom calcifications were stationary, homogenous cylinders. Fourth, we included a rather small patient population. Finally, we present first-experience data on CAC scoring using a commercially available dual-source PCD-CT. Hence, more information should be gathered in the future with larger studies.

In conclusion, our first phantom and in-vivo experience of CAC scoring on a clinical dual-source PCD-CT scanner suggests accurate CAC scoring using monoenergetic reconstructions, being constant across different radiation dose levels. Increasing strength levels of QIR and increasing VMI energy levels decrease CAC scores.

## Figures and Tables

**Table 1:** Patient baseline and scan characteristics.

Patient and Scan Characteristics (*n* = 20)	
Females	*n* = 12 (60%)
Age (years)	69 ± 12
Height (cm)	168 ± 12
Weight (kg)	76 ± 17
Body mass index (kg/m^2^)	27.0 ± 4.7
Tube current time product (mAs)	18 ± 6
Volume CT dose index (mGy)	3.3 ± 1.3
Dose length product (mGy × cm)	48 ± 19
Effective dose * (mSv)	0.67 ± 0.26

* a conversion factor of 0.014 mSv/mGy × cm was applied to calculate effective dose [16].

**Table 2:** Distribution of Agatston scores in the patients.

VMI/T3D	FBP/no QIR	QIR 1	QIR 2	QIR 3	QIR 4
60 keV	107 (48–1060)	100 (44–1026)	91 (40–999)	86 (36–974)	80 (33–952)
65 keV	105 (44–1024)	98 (40–989)	90 (36–957)	82 (33–930)	74 (30–896)
70 keV	86 (38–978)	76 (35–939)	65 (31–886)	64 (27–877)	59 (24–850)
75 keV	64 (29–895)	59 (26–867)	56 (23–839)	53 (21–819)	49 (20–803)
T3D	101 (46–1035)	95 (43–1013)	86 (39–981)	80 (37–959)	76 (34–934)

## Data Availability

Upon reasonable request to the corresponding author.

## Abbreviations

CAC: coronary artery calcium; EBCT, electron-beam CT; ECG, electrocardiogram; EID-CT, energy-integrating detector CT; FBP, filtered back projection; IQ, image quality; IQR, inter-quartile range; IRB, institutional review board; PCD-CT, photon-counting detector CT; virtual monoenergetic image, VMI.
